# Oxidative Stress and Fetal Growth Restriction Set Up Earlier in Undernourished Sheep Twin Pregnancies: Prevention with Antioxidant and Nutritional Supplementation

**DOI:** 10.3390/antiox11071287

**Published:** 2022-06-28

**Authors:** Víctor H. Parraguez, Francisco Sales, Oscar Peralta, Monica De los Reyes, Antonio Gonzalez-Bulnes

**Affiliations:** 1 Faculty of Veterinary Sciences, University of Chile, Santiago 8820808, Chile; operalta@uchile.cl (O.P.); mdlreyes@uchile.cl (M.D.l.R.); 2 Faculty of Agricultural Sciences, University of Chile, Santiago 8820808, Chile; 3 INIA-Kampenaike, Punta Arenas 6212707, Chile; fsales@inia.cl; 4 Faculty of Veterinary Sciences, Universidad CEU Cardenal Herrera, C/Tirant lo Blanc, 46115 Alfara del Patriarca, Valencia, Spain; antonio.gonzalezbulnes@uchceu.es

**Keywords:** fetal growth, nutrition, antioxidants, oxidative stress, sheep

## Abstract

Hypoxemia and oxidative stress, resulting in intrauterine growth restriction (IUGR) in undernourished twin sheep pregnancies, has been described in near-term studies. Our aim was to evaluate if the counteractive effects of maternal nutritional or antioxidant supplementation on the fetal redox status were evident before the accelerated fetal growth phase. Forty twin-bearing ewes grazing on natural Patagonian prairie were randomly assigned to four groups (*n* = 10 each; P: control ewes consuming mainly natural pasture; P+A: pasture plus antioxidants; P+C: pasture plus concentrate; P+A+C: pasture plus antioxidants and concentrate). Daily herbal antioxidants were supplemented in a feedstuff concentrate as a premix from day 35 until day 100 of gestation, when fetal venous cord blood samples and biometric measurements were obtained via cesarean section. The fetuses from group P were clearly hypoxemic. An analysis of variance showed that maternal antioxidant supplementation showed a trend of increased PO_2_, SatHb, and Ht, effects not observed in P+C fetuses. Antioxidants decreased the fetal MDA concentration (*p* < 0.05). Fetal TAC was increased by the antioxidants and concentrate (*p* < 0.05). Antioxidant supplementation showed a trend to increase fetal body weight but not biometry. The results suggest that negative effects of oxidative stress occur earlier than the overt growth arrest, and the maternal administration of antioxidants may constitute a good nutritional strategy for the early prevention of IUGR.

## 1. Introduction

In sheep, pregnancies developed under adverse conditions may lead to intrauterine growth restriction (IUGR) and fetal programming, which may have serious consequences for postnatal life and wellbeing [1]. Among these conditions, maternal undernutrition and multiple gestations play a critical role as main factors determining IUGR and, consequently, low birth weight [2]. Although the increase in prolificacy is considered a good strategy to increase productivity in grazing sheep systems, it leads to an increase in mortality due to low birth weight [3,4].

Sheep farming is developed either in business systems or as subsistence farming in small herds. Regardless of the above systems, the largest numbers of sheep are raised in territories of Asia and Africa [5], with little supply of forage being very common and, in many cases, the overloading of grasslands. Hence, undernutrition is a common breeding condition for sheep flocks [6]. Undernutrition is also a common condition in other territories, such as south American Patagonia. In Chile, the Patagonian steppe maintains more than 56% of the national sheep stock, where climatic conditions are extreme, and the natural grassland does not cover the nutritional requirements of the sheep, especially during pregnancy [7]. Thus, pregnancies develop under natural chronic undernutrition, with IUGR associated with near-term fetal hypoxemia and oxidative stress, effects that are greater in twin compared to single pregnancies [8]. This has potentially negative effects on the animals’ wellbeing and on the productive and economic outputs for breeders because low-birth-weight individuals often have higher mortality, lower postnatal growth and feed efficiency, poorer carcass conformation, lower meat yield and quality, delayed sexual development, and lower fertility than their normal-weight counterparts [9,10,11,12].

Many physiological parameters involved in animal reproduction have important roles in the productivity of farm animals. Advances in reproduction research studies have enabled the identification of strategies to improve the reproductive and productive capacity in livestock. Diet composition improvement represents a key factor to enhance the health status and welfare of animals [13], as well as to enhance productivity in livestock [14,15,16,17]. We recently showed that antioxidant supplementation during the entire gestation period in naturally undernourished ewes kept in Chilean Patagonia counteracted oxidative stress and increased fetal weight near lambing (day 140 of gestation; length of gestation = 148 days) in both single and twin pregnancies [18]. Furthermore, in a very recent study carried out in our undernourished twin pregnancy model in which the effects of nutritional supplementation (to cover the requirements of pregnancy) and antioxidant supplementation were compared, we found that both types of supplementation significantly improved the lamb birth weight [19]. In the present pilot study, we aim to contribute to the knowledge of the course of such effects, evaluating the redox status of twin fetuses and verifying whether the effects of nutritional and antioxidant supplementation are already evident at the end of the second term of gestation before starting the accelerated fetal growth.

## 2. Materials and Methods

### 2.1. Animals and Experimental Procedure

This pilot study was carried out at the INIA Kampenaike Research Farm, 60 km north of Punta Arenas, Chile (Chilean Patagonia; lat. 52°41′ S, long. 70°54′ W). Corriedale ewes (4–6 years old) from a commercial flock were synchronized using a CIDR (CIDR G^®^, Pfizer, Región Metropolitana, Chile) during 12 days, followed by a 300 IU eCG (Novormon^®^, Syntex, Provincia de Buenos Aires, Argentine) i.m. injection. Mating was carried out using fertile-proven rams whose chests were coated with a solution containing food-grade oil and colored earth. The exact day of mating was identified by daily visual inspection of the ewes’ colored rumps. Ultrasound examination was performed 30 days after mating, where 40 twin-bearing ewes were selected. The body weight (BW) and body condition scores (BCS, 1-to-5 scale [20]) were similar for all the groups (64.2 ± 1.0 Kg and 2.62 ± 0.10, respectively) at the start of the experiment. The pregnant ewes were randomly assigned into 4 equal-sized groups (*n* = 10 each) and ear-tagged with different colors according to each treatment. The groups were: P, control ewes consuming mainly natural pasture; P+A, ewes consuming natural pasture plus antioxidant supplementation; P+C, ewes consuming natural pasture plus concentrate supplementation; P+A+C, ewes consuming natural pasture plus antioxidant and concentrate supplementation. The animals were maintained under common grazing conditions on a representative Patagonian prairie *(Festuca gracillima*-*Chiliotrichium diffusum*; crude protein: 3.3%; metabolizable energy: 1.9 Mcal/kg; total digestible nutrients: 45%) with a stocking rate of 0.9 ewes per hectare and a dry matter availability of ~525 kg per hectare. Drinking water was offered ad libitum.

Supplementation with herbal antioxidants was performed by including a diet supplemented with herbal-based products with polyphenols mimicking vitamin C and E antioxidant activity (C-Power^®^ and Herbal-E50^®^, respectively; Nuproxa, Etoy, Switzerland) included as a premix (290 mg of each herbal antioxidant supplement per kg of diet). C-Power^®^ contains *Emblica officinalis* (containing mainly gallic acid) and *Ocimum sanctum* (containing apigenin), while Herbal-E50^®^ contains *Ocimum sanctum*, *Ocimum basilicum* (containing eugenol), and *Phyllanthus emblica* (containing gallic and tannic acids). C-Power^®^ and Herbal-E50^®^ (Nuproxa, Switzerland) were included as a premix in commercial feedstuff concentrate (CP: 17.0%; ME: 3.0 Mcal/kg) in doses of 10 and 7 g per Kg, respectively, dosing that was previously demonstrated to significantly prevent fetal oxidative stress and intrauterine restriction at term [19]. Ewes from the control group (P) received, daily, 50 g of concentrate without antioxidants, equaling the nutritional offer of the P+A group. Groups with nutritional supplementation (P+C and P+A+C) initially received 450 g of the same concentrate formulation, but without antioxidant inclusion. Supplementation began at 35.1 ± 0.1 days of gestation and continued until the end of the experiment at day 100 of gestation. The amount of concentrate offered was adjusted monthly according to the expected weight gain for each stage of gestation [21]. The concentrate intake was individual and was assessed daily. No refusals were observed for any of the treatment groups. Maternal BW was assessed monthly, while BCS was evaluated at the begin and at the end of the study.

Fetal sampling was carried out on day 100 of gestation (about ~67% of the total length of ovine pregnancy). A cesarean section was performed under spinal anesthesia through the administration of 2 mL 2% lidocaine hydrochloride (Lidocalm^®^, Drag Pharma, Santiago, Chile). Blood samples were drawn with heparinized syringes (1000 IU mL solution) via the umbilical vein of each fetus after the incision of the pregnant uterine horns. Simultaneously, a maternal carotid arterial blood sample was also drawn. One sample of fetal blood (1 mL) and the maternal sample were used for direct and immediate evaluation of the oxygenation status; a second fetal sample (5 mL) was centrifuged at 1200× *g* for 5 min at 4 °C, and the plasma was harvested and stored in liquid nitrogen until assayed for oxidative stress biomarkers. The fetuses were removed from the maternal uterus and immediately euthanized by barbiturate overdose (Opet^®^, Pro-Vet, Santiago, Chile). Due to the regulations of the experimental station in order to prevent the disposal of animals by euthanasia, the sheep were subjected to hysterorrhaphy, followed by suturing of the abdominal muscle layer and skin, to be taken to recovery, where they were administered analgesics (sodium metamizole, 40 mg Kg every 12 h, i.m.; Drag Pharma, Chile) and antibiotics (dihydrostreptomycin and penicillin in association, 5 mL per animal every 72 h; Combiótico^®^ L.A., Agrovet, Región Metropolitana, Chile). After 6 days of prophylactic treatment and observation, the sheep were returned to the herd. The fetal sex, BW, crown–rump length (CRL), thorax perimeter (TP), fore-limb (FL) and hind-limb (HL) lengths, and weights of the main internal organs and semitendinosus muscle for each individual fetus were registered.

### 2.2. Assessment of Maternal and Fetal Oxygenation Status

The partial pressure of oxygen (PO_2_), partial pressure of carbon dioxide (PCO_2_), Hb saturation by oxygen (SatHb), hematocrit (Ht), and pH values were measured in the umbilical vein and maternal arterial blood using an IL Synthesis 25TM gas analyzer (Instrumentation Laboratory, Lexington, MA, USA) adjusted to ovine body temperature [22]. 

### 2.3. Assessment of Oxidative Stress Biomarkers

The fetal redox status was evaluated by measurement of the plasma malondialdehyde (MDA) concentration and total antioxidant capacity (TAC). The quantifications of MDA and TAC in the plasma samples were performed using a TBARS colorimetric kit (TCA method, Assay Kit, Cayman Chemical Company, Ann Arbor, MI, USA) and an Antioxidant Assay Kit (Cayman Chemical Company, Ann Arbor, MI, USA), respectively. The assays were carried out according to the manufacturer’s instructions, and the absorbance levels were read at 540 and 405 nm, respectively, with a microplate reader (Perlong DNM-9602, Nanjing Perlove Medical Equipment Co. Ltd., Nanjing, China). Both assay kits have been previously proved with ovine plasma [18,19,23].

### 2.4. Statistical Analysis

Comparisons among the groups were performed with analysis of variance, using the general linear model procedure of SAS (GLM; SAS Institute Inc., Cary, NC, USA) after testing the data for normality. The maternal BW and BCS were analyzed through repeated measures analysis, considering pregnancy nutritional plan (only grazing or grazing plus concentrate feedstuff) and antioxidant supplementation (with or without) as independent effects. The fetal data were analyzed using a linear model including the fixed effects of maternal nutritional plan and antioxidant supplementation, as well as their interactions. The effects of fetal sex and the interactions of treatment and sex were not significant (*p* > 0.05) and were, therefore, removed from the model. Differences were considered significant when *p* < 0.05 and as a tendency when *p* < 0.1. When the results were significant, Duncan’s post hoc test was performed to establish which groups were different. The results were expressed as means ± SEM.

## 3. Results

### 3.1. Maternal Weight and Body Condition

**Figure 1:**
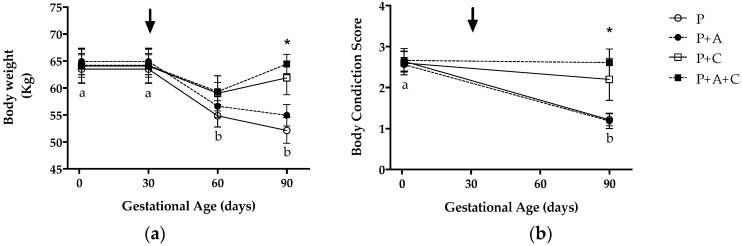
Mean (±SEM) body weight (**a**) and body condition score (**b**) from mating to day 90 of pregnancy in twin-bearing ewes maintained under extensive Magellan conditions, consuming only natural pasture (group P), natural pasture plus antioxidant supplementation (group P+A), natural pasture plus concentrate supplementation (group P+C), and natural pasture plus antioxidant and concentrate supplementation (group P+A+C). The arrow shows the time when antioxidant or nutritional supplementation began. Asterisks indicate significant differences between sheep supplemented and nonsupplemented with concentrate at the same sampling time (*p* < 0.01). Different letters below the symbols indicate significant differences between gestational ages (0–30 and 60–90) in groups without concentrate (*p* < 0.01).

The ewes’ BW (Figure 1a) was significantly affected by the time of pregnancy in the groups without concentrate supplementation (P and P+A; *p* < 0.001). Supplementation with antioxidants had no effect on BW (*p* > 0.05). A significant decrease in the ewes’ BW was observed from 60 days of gestation in the groups maintained only on grazing with concentrate (*p* < 0.01). This decrease was accentuated as gestation proceeded. The groups receiving concentrate showed a small but nonsignificant decrease in body weight at 60 days of pregnancy, but with a clear recovery at 90 days. A clear divergence in ewe BW behavior between the groups that received and did not receive concentrate was observed from 60 days of gestation, reaching statistical significance at day 90 (*p* < 0.01). Maternal BCS (Figure 1b) was also affected by the time of gestation in groups without concentrate, being significantly less at day 90 (*p* < 0.01). No changes in BCS were observed in the groups supplemented with concentrate (*p* > 0.05). 

### 3.2. Effects of Maternal Antioxidant and Nutritional Supplementation on Maternal and Fetal Blood Gases

The maternal arterial gases were not affected by the treatments; therefore, the average values obtained from all of the ewes were: PO_2_ 88.5 ± 2.3 mm Hg; PCO_2_ 40.2 ± 0.1 mm Hg; SatHb 93.3 ± 0.9%; Ht 30.5 ± 0.9; and pH 7.44 ± 0.01.

The blood variables associated with oxygen transportation in the fetuses are shown in Table 1. Fetuses from mothers kept only by consuming natural pasture during pregnancy (group P) were clearly hypoxemic with very low PO_2_ and SatHb values. Maternal antioxidant supplementation showed a trend to increase PO_2_, SatHb, and Ht (*p* = 0.066). No effect of the antioxidants was observed for the PCO_2_ or for pH (*p* > 0.05). In contrast with the effect of maternal antioxidants, concentrate supplementation had no effect on fetal PO_2_, SatHb, and Ht (*p* > 0.05), but it increased the PCO_2_ and the pH (*p* < 0.05). No interactions between the antioxidants and nutritional plans were observed.

### 3.3. Effects of Maternal Antioxidant and Nutritional Supplementation on Fetal Traits

#### 3.3.1. Effects on Fetal Oxidative Status

**Figure 2:**
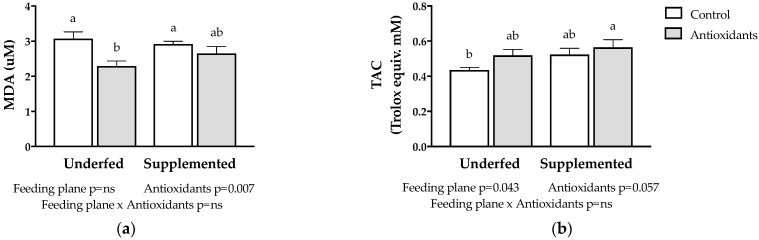
Mean (±SEM) plasma MDA (**a**) and TAC (**b**) in twin lamb fetuses at 100 days of gestational age whose mothers were maintained under extensive Patagonian conditions, consuming only natural pasture (underfed groups) or natural pasture plus concentrate supplementation (supplemented groups). The empty bars correspond to animals not receiving antioxidants (control). The gray bars correspond to animals receiving antioxidants (antioxidants). The corresponding effects and their statistical significances are presented as footnotes in each figure. MDA: malondialdehyde; TAC: total antioxidant capacity. Different letters on the bars indicate significant differences (Duncan’s post hoc test).

The biomarkers of oxidative stress in the fetal plasma are shown in Figure 2. The administration of antioxidants to the pregnant ewes significantly decreased the fetal concentrations of MDA (*p* = 0.007; Figure 2a), with an effect of greater magnitude in the group that did not receive concentrate (underfed, group P+A). The effect of antioxidants on fetal TAC was almost significant (*p* = 0.067; Figure 2b), increasing its value independently of the feeding plan. The effect of antioxidants was higher in the underfed group (P+A), as in the case of MDA. The feeding plan had no effect on the plasma concentrations of MDA (*p* > 0.05; Figure 2a), while it was significant for plasma TAC (*p* < 0.05; Figure 2b). In the latter case, supplementation with concentrate increased the TAC of the fetuses (groups P+C and P+A+C). 

#### 3.3.2. Effects on Fetal Body Weight and Biometry

The effects of maternal supplementation with antioxidants or concentrated feedstuff on different biometric variables from the twin fetuses in a model of natural, underfed pregnancy are presented in Table 2. Antioxidant supplementation showed a trend to increase fetal BW (*p* = 0.068), but no other effects were observed (*p* > 0.05). In contrast, concentrate supplementation affected all the fetal traits evaluated (*p* < 0.055), leading to an increase in the magnitude of each trait. No interaction between antioxidant and concentrate supplementation was obtained (*p* > 0.05).

#### 3.3.3. Effects on Fetal Semitendinosus Muscle and Main Organs

Maternal supplementation with antioxidants had no effect on the weight of the fetal semitendinosus muscle or on the organs evaluated (Table 3; *p* > 0.05). In contrast, maternal supplementation with concentrate had significant effects on all of the evaluated fetal organs (*p* < 0.01), with exception of the brain (*p* > 0.05). Thus, the semitendinosus muscle, kidneys, brown adipose tissue, heart, and liver were heavier in the groups receiving concentrate (P+C and P+A+C). For these variables, there was also no interaction between the effects of both types of supplementation.

## 4. Discussion

The present pilot study supported previous evidence on the state of the subnutrition of pregnant sheep maintained with natural grazing [5,6,7,8] (a state worsened in case of twin pregnancies [8,18]), as addressed in the present study by the finding of both decreasing BW and BCS in the sheep throughout pregnancy. Undernutrition during pregnancy has obvious consequences on the fetal growth, birth weight, and postnatal phenotype and performance of the progeny, as has previously been reported [9,10,11,12]. Thus, the breeding of sheep in natural rangelands, a main economic resource in many regions of the world, should include nutritional supplementation. In this sense, the results of our study also supported previous data proving that supplementation with concentrate maintains maternal BW and BCS at levels that are sufficient for adequate fetal growth and birth weight [18,24]. However, sheep raised in natural rangelands generally do not receive concentrates since, usually, these are not widely available and are, therefore, too expensive. Hence, alternative strategies that can improve the productive outputs of animals reared in these conditions without a strong outlay are mandatory.

Fetal growth restriction is known to be primarily related to impaired supplies of nutrients and oxygen and, consequently, a weakened antioxidant defense system [25,26,27,28]. We have shown in previous studies that the deleterious effects of the oxidative status at the fetoplacental unit can be prevented by the administration of antioxidant agents (e.g., antioxidant vitamins), which ameliorate the antioxidant/oxidative ratio, improve the placental function, and increase the weight and viability of the newborn [29,30]. The same results have been found when applying antioxidant supplementation to underfed grazing ewes [18,19], but the present study supported that the negative effects of oxidative stress and, thus, the positive effects of antioxidants, are present earlier, even prior to the last term of pregnancy (i.e., prior to the overt growth arrest that becomes apparent between 120 and 130 days of ovine underfed pregnancies [31]), which has been traditionally identified as the moment in which fetal growth retardation is established.

In this sense, in the present trial performed at day 100 of gestation (about 67% of the total length of ovine pregnancy), we found growth retardation in underfed twin fetuses. A previous study showed that a divergence in fetal weight between singles and twins could be detected before day 100 of gestation [32]. Our data support previous evidence of earlier fetal growth retardation, with still a slight and preliminary brain-sparing effect, in underfed twin pregnancies at day 115 (around 75% of the total length of ovine pregnancy) [33]. Concomitantly, the current results evidenced that fetuses had a hypoxemic state with very low PO_2_ and SatHb values and a decreased TAC early in these pregnancies, before starting accelerated fetal growth. The growth retardation, hypoxia, and oxidative stress in these fetuses were compensated by maternal supplementation with either concentrate or antioxidants.

Maternal supplementation with concentrate in twin pregnancies, in agreement with previous studies [17,24], increased the body weight and size of the fetuses, as well as the weights of all their organs, except the brain, which may be related to evidence that a brain-sparing effect is established later [32]. Interestingly, the semitendinosus muscle weight increased in concentrate-supplemented animals, and this could have beneficial effects on fetal growth, as lower fetal weights in twins have been associated with reduced weights in muscle [34,35]. On the other hand, maternal antioxidant supplementation showed only a trend to increase fetal BW, with no effects in organ weights and no interaction with concentrate supplementation.

Maternal supplementation with antioxidants without modifying maternal arterial gases showed a trend to increase the PO_2_, SatHb, and Ht of the fetuses, an effect that was not found in case of maternal supplementation with concentrate. The feeding plan had no effect on maternal and fetal blood gases or fetal plasma concentrations of MDA, but it increased the TAC of the fetuses, supporting the narrow relationship between the fetal growth pattern and antioxidant defense system that has been previously reported [25,26,27,28]. The administration of antioxidants to pregnant ewes also showed a trend for increasing fetal TAC and significantly decreased the MDA in both concentrate-supplemented or unsupplemented pregnancies, the effect being higher in the underfed group.

These results indicated that the effect of antioxidants on plasma parameters is direct and independent of the effects on fetal growth. Such hypotheses are supported by previous data addressing that antioxidants have increased umbilical flow, which favored fetal growth [36], and placental angiogenesis, which favored placental function and efficiency and, in turn, fetal growth [29,37,38]. Moreover, the assessment of the placental transfer of vitamins C and E orally administered to pregnant sheep was associated with increased fetal cord levels of both vitamins, resulting in an improved antioxidant status and enhanced fetal growth with increased placental efficiency [39]. However, this trial was performed at a later pregnancy stage (140 days of gestation; around 95% of the total length of ovine pregnancy). In the present study, the effects of nutritional and antioxidant supplementation were already evident at the end of the second term of gestation, before starting accelerated fetal growth. From previous data, we can hypothesize that these events may be related to early alterations in placental development and function, as suggested by our previous study [18]. In fact, inappropriate maternal nutrition during early and mid-pregnancy can significantly disrupt placental development, which reaches a maximum growth by approximately days 75–80 of gestation [39].

## 5. Conclusions

The results of the present study indicated that negative effects of oxidative stress, as well as its relationship with fetal growth retardation, in compromised, underfed, twin pregnancies were present at stages earlier than the overt growth arrest, which becomes apparent at the last month of gestation. Hence, strategies for increasing the prolificacy and productivity of herds under natural rangeland systems by increasing twin-bearing ewes may be penalized at early pregnancy stages, and such an event should, therefore, be counteracted early during pregnancy. The present results supported that, in addition to nutritional supplementation, the administration of herbal antioxidants may constitute a good nutritional strategy for sheep reared in harsh environmental conditions.

## Figures and Tables

**Table 1:** Fetal blood gases at 100 days of gestation in underfed twin sheep pregnancies: effects of maternal antioxidant and nutritional supplementation.

		Group			*p*-Value		
Blood Trait	P	P+A	P+C	P+A+C	AOX	FP	AOXxNP
PO_2_ (mm Hg)	17.4 ± 0.9	22.5 ± 2.3	21.2 ± 2.1	21.7 ± 1.7	0.071	ns	ns
PCO_2_ (mm Hg)	57.9 ± 1.4	55.5 ± 2.2	45.6 ± 2.4	48.8 ± 1.6	ns	<0.001	ns
SatHb (%)	18.8 ± 1.8	29.6 ± 4.4	28.3 ± 4.8	29.7 ± 4.2	0.066	ns	ns
Ht (%)	36.3 ± 1.2	37.8 ± 1.0	35.9 ± 0.8	37.2 ± 0.8	0.060	ns	ns
pH	7.33 ± 0.02	7.31 ± 0.02	7.34 ± 0.02	7.36 ± 0.02	ns	0.043	ns

PO_2_: partial pressure of oxygen; PCO_2_: partial pressure of carbon dioxide; SatHb: saturation of hemoglobin by oxygen; Ht: hematocrit; P: control ewes consuming mainly natural pasture; P+A: ewes consuming natural pasture plus antioxidant supplementation; P+C: ewes consuming natural pasture plus concentrate supplementation; P+A+C: ewes consuming natural pasture plus antioxidant and concentrate supplementation; AOX: antioxidants; FP: feeding plan; ns: not significant.

**Table 2:** Fetal body weight and biometry at 100 days of gestation in underfed twin sheep pregnancies: effects of maternal antioxidant and nutritional supplementation.

		Group			*p*-Value		
Blood Trait	P	P+A	P+C	P+A+C	AOX	FP	AOXxNP
BW (g)	869 ± 28 ^c^	915 ± 26 ^bc^	976 ± 33 ^ab^	1050 ± 35 ^a^	0.068	<0.001	ns
CRL (cm)	26.2 ± 0.4 ^b^	26.6 ± 0.3 ^b^	27.0 ± 0.5 ^ab^	27.8 ± 0.4 ^a^	ns	0.016	ns
TP (cm)	20.1 ± 0.3 ^b^	20.4 ± 0.2 ^b^	20.8 ± 0.3 ^ab^	21.3 ± 0.3 ^a^	ns	0.008	ns
FL (cm)	16.3 ± 0.3 ^b^	16.4 ± 0.3 ^b^	17.2 ± 0.4 ^a^	17.2 ± 0.3 ^a^	ns	0.055	ns
HL (cm)	18.7 ± 0.3 ^c^	18.9 ± 0.2 ^bc^	19.7 ± 0.5 ^ab^	19.8 ± 0.3 ^a^	ns	0.003	ns

BW: body weight; CRL: crown–rump length; TP: thorax perimeter; FL fore-limb length; HL: hind-limb length; P: control ewes consuming mainly natural pasture; P+A: ewes consuming natural pasture plus antioxidant supplementation; P+C: ewes consuming natural pasture plus concentrate supplementation; P+A+C: ewes consuming natural pasture plus antioxidant and concentrate supplementation; AOX: antioxidants; FP: feeding plan. Different superscript letters indicate significant differences among groups (Duncan’s post hoc test); ns: not significant.

**Table 3:** Fetal semitendinosus muscle and main organ weights at 100 days of gestation in underfed twin sheep pregnancies: effects of maternal antioxidant and nutritional supplementation.

		Group			*p*-Value		
Fetal Trait	P	P+A	P+C	P+A+C	AOX	FP	AOXxNP
SM (g)	1.80 ± 0.06 ^c^	1.91 ± 0.1 ^bc^	2.08 ± 0.07 ^ab^	2.19 ± 0.08 ^a^	ns	0.003	ns
Kidneys (g)	4.80 ± 0.13 ^b^	4.96 ± 0.14 ^b^	5.14 ± 0.14 ^ab^	5.43 ± 0.16 ^a^	ns	0.009	ns
BAT (g)	2.33 ± 0.20 ^bc^	2.09 ± 0.15 ^c^	2.72 ± 0.20 ^b^	3.34 ± 0.23 ^a^	ns	<0.001	ns
Heart (g)	7.98 ± 0.32 ^ab^	7.58 ± 0.32 ^b^	8.78 ± 0.53 ^a^	8.69 ± 0.23 ^a^	ns	0.007	ns
Brain (g)	23.5 ± 0.76	23.2 ± 0.53	22.3 ± 0.70	23.5 ± 0.66	ns	ns	ns
Liver (g)	45.1 ± 1.74 ^bc^	42.8 ± 1.61 ^c^	50.1 ± 1.88 ^b^	56.4 ± 1.79 ^a^	ns	<0.001	ns
Brain/Liver	0.53 ± 0.02 ^a^	0.55 ± 0.03 ^a^	0.45 ± 0.02 ^b^	0.42 ± 0.01 ^b^	ns	<0.001	ns

SM: semitendinosus muscle; BAT: brown adipose tissue; P: control ewes consuming mainly natural pasture; P+A: ewes consuming natural pasture plus antioxidant supplementation; P+C: ewes consuming natural pasture plus concentrate supplementation; P+A+C: ewes consuming natural pasture plus antioxidant and concentrate supplementation; AOX: antioxidants; FP: feeding plan. Different superscript letters indicate significant differences among groups (Duncan’s post hoc test); ns: not significant.

## Data Availability

Data is contained within the article.

## References

[1] González-Bulnes A., Parraguez V.H., Berlinger F., Barbero A., García-Contreras C., Lopez-Tello J., Pesantez-Pacheco J.L., Martínez-Ros P. (2020). The impact of prenatal environment on postnatal life and performance: Future perspectives for prevention and treatment. Theriogenology.

[2] McCoard S.A., Sales F.A., Sciascia Q.L. (2017). Invited review: Impact of specific nutrient interventions during mid-to-late gestation on physiological traits important for survival of multiple-born lambs. Animal.

[3] McCoard S. (2017). Issues and opportunities to capitalize on increased litter size in hill country sheep farming systems—A New Zealand perspective. Anim. Front..

[4] Hinch G.N., Brien F. (2014). Lamb survival in Australian flocks: A review. Anim. Prod. Sci..

[5] Skapetas B., Kalaitzidou M. (2017). Current Status and Perspectives of Sheep Sector in the World. Livestock Res. Rur. Dev..

[6] Tibbo M., Philipsson J., Ayalew W. Sustainable Sheep Breeding Programmes in the Tropics: A Framework for Ethiopia. Proceedings of the Conference on International Agricultural Research for Development, University of Bonn.

[7] Sales F., Strauch O. Efecto del tipo de preñez sobre la variación de peso invernal en ovejas Corriedale. Proceedings of the XXXI Reunión Anual de la Sociedad Chilena de Producción Animal, Centro Regional de Investigación, INIA.

[8] Sales F., Peralta O.A., Narbona E., McCoard S., De los Reyes M., González-Bulnes A., Parraguez V.H. (2018). Hypoxia and oxidative stress are associated with reduced fetal growth in twin and undernourished sheep pregnancies. Animals.

[9] Zambrano E., Guzmán C., Rodríguez-González G.L., Durand-Carvajal M., Nathanielsz P.W. (2014). Fetal programming of sexual development and reproductive function. Mol. Cell. Endocrinol..

[10] McCoard S., Koolaard J., Charteris A., Luo D. Brief communication: Effect of twinning and sex on carcass weight and composition in lambs. Proceedings of the New Zealand Society of Animal Production.

[11] Nash M.L., Hungerford L.L., Nash T.G., Zinn G.M. (1996). Risk factors for perinatal and postnatal mortality in lambs. Vet. Rec..

[12] Greenwood P.L., Cafe L.M., Hearnshaw H., Hennessy D.W., Thompson J.M., Morris S.G. (2006). Long-term consequences of birth weight and growth to weaning on carcass, yield and beef quality characteristics of Piedmontese- and Wagyu-sired cattle. Aust. J. Exp. Agric..

[13] Abbate J.M., Macrì F., Capparucci F., Iaria C., Briguglio G., Cicero L., Salvo A., Arfuso F., Ieni A., Piccione G. (2020). Administration of protein hydrolysates from anchovy (*Engraulis encrasicolus*) waste for twelve weeks decreases metabolic dysfunction-associated fatty liver disease severity in ApoE-/-mice. Animals.

[14] Vazzana I., Rizzo M., Giambelluca S., Zumbo A., Piccione G., Monteverde V. (2014). The response of some blood constituents after administration of two different diets in goats. Comp. Clin. Pathol..

[15] Avondo M., Pagano R., Guastella A., Criscione A., Di Gloria M., Valenti B., Piccione G., Pennisi P. (2009). Diet selection and milk production and composition in Girgentana goats with different αs1-casein genotype. J. Dairy Res..

[16] Armato L., Gianesella M., Morgante M., Fiore E., Rizzo M., Giudice E., Piccione G. (2016). Rumen volatile fatty acids × dietary supplementation with live yeast and yeast cell wall in feedlot beef cattle. Acta Agric. Scand..

[17] Monteverde V., Congiu F., Vazzana I., Dara S., Di Pietro S., Piccione G. (2017). Serum lipid profile modification related to polyunsaturated fatty acid supplementation in thoroughbred horses. J. Appl. Anim. Res..

[18] Sales F., Peralta O.A., Narbona E., McCoard S., Lira R., De los Reyes M., González-Bulnes A., Parraguez V.H. (2019). Maternal supplementation with antioxidant vitamins in sheep results in increased transfer to the fetus and improvement of fetal antioxidant status and development. Antioxidants.

[19] Parraguez V.H., Sales F., Peralta O.A., Narbona E., Lira R., De los Reyes M., González-Bulnes A. (2020). Supplementation of underfed twin-bearing ewes with herbal vitamins C and E: Impacts on birth weight, postnatal growth, and pre-weaning survival of the lambs. Animals.

[20] Jefferies B.C. (1961). Body condition scoring and its use in management. Tasm. J. Agric..

[21] Van der Linden D., Kenyon P., Jenkinson C.M.C., Peterson S., Blair H.T. (2011). Carry-over effects of ewe nutrition and birth rank during the previous pregnancy on the milking performance during the subsequent lactation of Romney ewes. Anim. Prod. Sci..

[22] (2018). Merck Veterinary Manual. http://www.merckvetmanual.com.

[23] Cofré E., Peralta O.A., Raggi A., De los Reyes M., Sales F., González-Bulnes A., Parraguez V.H. (2017). Ram semen deterioration by short-term exposure to high altitude is prevented by improvement of antioxidant status. Animal.

[24] Cranston L., Kenyon P.R., Corner-Thomas R.A., Morris S.T. (2017). The potential interaction between ewe body condition score and nutrition during very late pregnancy and lactation on the performance of twin-bearing ewes and their lambs. Asian-Australas. J. Anim. Sci..

[25] Biri A., Bozkurt N., Turp A., Kavutcu M., Himmetoglu O., Durak I. (2007). Role of oxidative stress in intrauterine growth restriction. Gynecol. Obstet. Investig..

[26] Gupta P., Narang M., Banerjee B.D., Basu S. (2004). Oxidative stress in term small for gestational age neonates born to undernourished mothers: A case control study. BMC Pediatr..

[27] Kamath U., Rao G., Kamath S.U., Rai L. (2006). Maternal and fetal indicators of oxidative stress during intrauterine growth retardation (IUGR). Indian J. Clin. Biochem..

[28] Li H.P., Chen X., Li M.Q. (2013). Gestational diabetes induces chronic hypoxia stress and excessive inflammatory response in murine placenta. Int. J. Clin. Exp. Pathol..

[29] Parraguez V.H., Atlagich M., Araneda O., Garcia C., Muñoz A., De los Reyes M., Urquieta B. (2011). Effects of antioxidant vitamins on newborn and placental traits in gestations at high altitude: Comparative study in high and low altitude native sheep. Reprod. Fertil. Dev..

[30] Parraguez V.H., Urquieta B., De los Reyes M., Gonzalez-Bulnes A., Astiz S., Muñoz A. (2013). Steroidogenesis in sheep pregnancy with intrauterine growth retardation by high-altitude hypoxia: Effects of maternal altitudinal status and antioxidant treatment. Reprod. Fertil. Dev..

[31] Symonds M.E., Budge H., Stephenson T., McMillen I.C. (2001). Fetal endocrinology and development-manipulation and adaptation to long-term nutritional and environmental challenges. Reproduction.

[32] Gootwine E., Spencer T.E., Bazer F.W. (2007). Litter-size-dependent intrauterine growth restriction in sheep. Animal.

[33] Barbero A., Porcu C., Spezzigu A., Succu S., Dattena M., Gallus M., Molle G., Naitana S., Gonzalez-Bulnes A., Berlinguer F. (2018). Changes in renal hemodynamics of undernourished fetuses appear earlier than IUGR evidences. J. Dev. Orig. Health Dis..

[34] McCoard S.A., Peterson S.W., McNabb W.C., Harris P.M., McCutcheon S.N. (1997). Maternal constraint influences muscle fiber development in fetal lambs. Reprod. Fertil. Dev..

[35] McCoard S.A., McNabb W.C., Peterson S.W., McCutcheon S.N., Harris P.M. (2000). Muscle growth, cell number, type and morphometry in single and twin fetal lambs during mid to late gestation. Reprod. Fertil. Dev..

[36] Thakor A.S., Herrera E., Giussani D.A., Serón-Ferré M. (2010). Melatonin and vitamin C increase umbilical blood flow via nitric oxide-dependent mechanisms. J. Pineal Res..

[37] Kasimanickam R., Kasimanickam V., Rodriguez J.S., Pelzer K., Sponenberg P., Thatcher C.D. (2010). Tocopherol induced angiogenesis in placental vascular network in late pregnant ewes. Reprod. Biol. Endocrinol..

[38] Richter H.G., Hansell J.A., Raut S., Giussani D.A. (2009). Melatonin improves placental efficiency and birth weight and increases the placental expression of antioxidant enzymes in undernourished pregnancy. J. Pineal Res..

[39] Osgerby J.C., Wathes D.C., Howard D., Gadd T.S. (2004). The effect of maternal undernutrition on the placental growth trajectory and the uterine insulin-like growth factor axis in the pregnant ewe. J. Endocrinol..

